# Nanoparticle Labeling of Bone Marrow-Derived Rat Mesenchymal Stem Cells: Their Use in Differentiation and Tracking

**DOI:** 10.1155/2015/298430

**Published:** 2015-01-14

**Authors:** Ece Akhan, Donus Tuncel, Kamil C. Akcali

**Affiliations:** ^1^Department of Molecular Biology and Genetics, Bilkent University, 06800 Ankara, Turkey; ^2^Department of Chemistry, Bilkent University, 06800 Ankara, Turkey; ^3^Department of Biophysics, Ankara University Faculty of Medicine, 06100 Ankara, Turkey

## Abstract

Mesenchymal stem cells (MSCs) are promising candidates for cellular therapies due to their ability to migrate to damaged tissue without inducing immune reaction. Many techniques have been developed to trace MSCs and their differentiation efficacy; however, all of these methods have limitations. Conjugated polymer based water-dispersible nanoparticles (CPN) represent a new class of probes because they offer high brightness, improved photostability, high fluorescent quantum yield, and noncytotoxicity comparing to conventional dyes and quantum dots. We aimed to use this tool for tracing MSCs' fate *in vitro* and *in vivo*. MSC marker expression, survival, and differentiation capacity were assessed upon CPN treatment. Our results showed that after CPN labeling, MSC markers did not change and significant number of cells were found to be viable as revealed by MTT. Fluorescent signals were retained for 3 weeks after they were differentiated into osteocytes, adipocytes, and chondrocytes *in vitro*. We also showed that the labeled MSCs migrated to the site of injury and retained their labels in an *in vivo* liver regeneration model. The utilization of nanoparticle could be a promising tool for the tracking of MSCs *in vivo* and *in vitro* and therefore can be a useful tool to understand differentiation and homing mechanisms of MSCs.

## 1. Introduction

Stem cells are the new hope for the century since they have the potential to differentiate into several types of cells and tissues. Applications involving the use of stem cells in humans that might have been considered “science fiction” fewer than 20 years ago are now being utilized with a great success rate [1, 2]. Described by the pioneering studies of Friedenstein et al. [3] in 1970, mesenchymal stem cells (MSCs) are multipotent cells, capable of self-renewal and differentiating into multiple lineages, such as osteocytes, adipocytes, chondrocytes, myoblasts, and cardiomyocytes [4–6]. MSCs have a high potential for regenerative medicine and tissue engineering not only due to their intrinsic self-renewal capacity and ability to differentiate functional cell types in specific tissues but also due to their homing capacity and nonimmunogenic features [2, 7]. Therefore use of MSCs may provide new strategies for many debilitating diseases including neurodegenerative and cardiovascular diseases, diabetes, and cancer [5, 8, 9].

One of the hurdles that need to be tackled is to be sure that transplanted stem cells are able to find the injured tissue and function. Tracking the stem cells* in vivo* and* in vitro* has beneficiary effects on understanding the biology of stem cells [10]. Several labeling agents are being used for tracking the MSCs and differentiated cells [10–12] and involve the use of fluorescence-based detection schemes [13, 14]. Small fluorescent dyes and fluorescent proteins are used as traditional fluorescent markers but exhibit poor photostability as they fade away rapidly during imaging [15] limiting their use in longer-term monitoring of live cells. Luminescent nanoparticles (NPs), such as quantum dots and dye-loaded silica NPs, appear to be more suitable for these purposes as these NPs possess high brightness and photostability compared to small fluorescent dyes [16, 17]. However, their cytotoxicity is considered a serious problem for* in vivo* application because of the presence of toxic heavy metals (e.g., cadmium). Dye-loaded silica NPs also have drawbacks since only limited amount of dyes can be loaded due to *π*-*π* interaction between the dye molecules, which causes reduced fluorescent quantum yields [18]. *π*-*π* interactions are noncovalent interactions in which electron rich *π* system can interact with a cationic or neutral metal, an anion, or another *π* system from another molecule. This type of noncovalent interactions involving *π* systems is known to have a paramount role in biological events such as protein-ligand recognition [19].

A largely unexplored alternative is the use of functional-conjugated polymer particles as fluorescent labels. Recently NPs based on conjugated polymers (CPN) are emerging as a new class of luminescent NPs [20]. These NPs have many potential applications, including imaging agents, biosensors, and optoelectronic devices, because of their high quantum yields, molar absorptivity, photostability, and easy synthesis [21, 22].

We aim to demonstrate the use of self-fluorescence CPN as a biocompatible photostable fluorescent label in order to follow the fate of MSCs* in vitro* and* in vivo*.

## 2. Material and Methods

### 2.1. Isolation and Culture of MSCs

The MSCs were obtained from three 6-month-old Sprague-Dawley female rats. After cervical dislocation, bone marrow heterogeneous cell population was obtained from femur and tibia by flushing DMEM (Dulbecco's modified Eagle's medium, Invitrogen, Paisley, UK) containing 10% FBS (fetal bovine serum, HyClone, Logan, UT, USA). The isolated MSCs were cultured in plastic culture dishes with MesenCult Media (StemCell Technology, Vancouver, Canada) including 20% supplement (StemCell Technology) and a 1% penicillin/streptomycin solution (HyClone). The cells were cultured in a 5% CO_2_ incubator at 37°C. The medium of cells was changed twice a week for 14 days.

### 2.2. Labeling MSCs with Conjugated Polymer Nanoparticles (CPNs)

CPNs were prepared at the Department of Chemistry at Bilkent University. The copolymer was synthesized using the Suzuki coupling polymerization technique and after the synthesis of polymers they were converted into nanoparticles by reprecipitation method as shown previously [22]. Media of MSCs on 13th day were changed with media including CPNs in 1 : 4 ratio (25 *μ*g/mL) of MesenCult media. MSCs were checked under fluorescence microscopy with fluorescein isothiocyanate (FITC) filter. 494 nm is the max-excitation and 519 nm is the max-emission of FITC. ImageJ analysis was performed to count CPN-MSCs in the slides.

### 2.3. Total RNA Isolation, RT-PCR

In order to assess the effect of CPN labeling in the expression of the markers of MSCs, we investigated their expression at day 14 of the culture. Cultured CPN treated and nontreated MSCs were trypsinized and total RNAs were isolated with the RNeasy Mini kit Stem Cell Rev and Rep (Qiagen, Hilden, Germany) according to the manufacturer's protocol. The cDNAs were synthesized from total RNA with the DyNAmo cDNA synthesis kit (Finnzymes, Espoo, Finland) according to the manufacturer's protocol. These cDNAs were used for MSCs marker analysis with PCR. Primers and PCR conditions used for cDNA amplification were listed in Tables 1 and 2.

### 2.4. MTT Assay for Cellular Metabolic Activity

The viability of CPN treated MSCs was detected via 3-[4,5-dimethylthiazol-2-yl]-2,5-diphenyl tetrazolium bromide (MTT) assay (Roche Molecular Biochemicals, Indianapolis, USA). 10^4^ MSCs were cultured in the wells of 96-well plates. Three sets of MSCs isolated from 6-month-old rat bone marrow samples were treated with MesenCult medium with CPNs and three sets were cultured only in MesenCult medium for 24 hours, at which time the MTT reagent was applied at a final concentration of 0.5 mg/mL for 4 hours at 37°C in a 5% CO_2_ incubator. The absorbance of the end product formazan was measured with ELISA reader (BioTek, KCJrWin software) at a primary wavelength of 551 nm and a maximum wavelength of 601 nm. The numeric values for the MTT assays were listed in Table 3.

### 2.5. *Lactate Dehydrogenase* (LDH) Assay for Cellular Toxicity

2 × 10^4^ MSCs were placed in 96-well plates in triplicate. CPN was added to the MSC culture media in 1 : 4 ratio (25 *μ*g/mL). Since 1% Triton-X is known to be toxic for cells, it was used as a positive control for LDH activity (high control) and hence maximum toxicity. We also used another control group (low control) in which MSCs were not labeled with CPN and incubated in the medium without Triton-X. This provided data for the minimum toxicity. After 24 h incubation, the toxicity of CPN was determined by measuring the release of cytoplasmic LDH from the damaged cells with Cytotoxicity Detection Kit (LDH, Clontech) according to manufacturer's protocol. The readings were performed by using an ELISA reader (BioTek, KCJrWin software) at the wavelength value of 490 nm. For the calculations, the average value of background group readings was subtracted from all of the sample readings. The percent cytotoxicity value was calculated by the following equation:

(1) Cytotoxicity  %=Triplicate  Absorbance−Low  ControlHigh  Control−Low  Control∗100.

The numeric values for the LDH assays were listed in Table 4.

### 2.6. Osteogenic Differentiation of CPN-Labelled MSCs and Alizarin Red Staining

On the 13th day of culture when MSCs reached 80% confluency, CPN was added to MesenCult media in 1 : 4 ratio (25 *μ*g/mL). After MSCs were incubated with CPN for 24 h, the medium was changed with freshly prepared osteogenic differentiation medium containing 0.1 *μ*M dexamethasone (Sigma), 0.2 mM ascorbic acid 2-phosphate (Sigma), and 10 mM glycerol-2-phosphate (Sigma) to LG-DMEM (low glucose Dulbecco's modified Eagle's medium) (HyClone), 1x penicillin-streptomycin (HyClone), and 10% FBS (HyClone). Cells were cultured for 21 days in a 5% CO_2_ incubator at 37°C. The MSCs were not further treated with CPN during differentiation. To assess the effect of CPN on osteogenic differentiation, cells were fixed with 10% formaldehyde for 15 minutes and were stained with Alizarin Red (Sigma) for 20 minutes to detect the presence of calcium node formation [9]. Then the cells were examined under bright-field microscopy. For CPN detection, same sections were examined under fluorescence microscope with FITC filter. For the evaluation, we used an excitation wavelength in the range of 450–500 nm and detection in the range of 515–565 nm. For counter staining to visualize nuclei, the specimens were mounted using UltraCruz (Santa Cruz) mounting medium with DAPI.

### 2.7. Adipogenic Differentiation of NP-Labelled MSCs and Oil Red O Staining

On the 13th day of culture when MSCs reached 80% confluency, CPN was added to MesenCult media in 1 : 4 ratio (25 *μ*g/mL). After MSCs were incubated with CPN for 24 h, the medium was changed with freshly prepared adipogenic differentiation media consisting of 1 *μ*M dexamethasone (Sigma), 10 *μ*g/mL insulin (Sigma), 100 *μ*M indomethacin (Sigma), and 0.5 mM IBMX (isobutylmethylxanthine) (Sigma) to LG-DMEM (HyClone), 1x penicillin-streptomycin (HyClone), and 10% FBS (HyClone). Cells were cultured for 21 days in a 5% CO_2_ incubator at 37°C. The MSCs were not further treated with CPN during differentiation. To assess the effect of CPN on adipogenic differentiation, cells were fixed with 4% paraformaldehyde and (Sigma) Oil Red O (Sigma) staining was used for detection of accumulated oil droplets [9]. The cells were then examined under bright-field microscopy. For CPN detection, the same sections were examined under fluorescence microscopy with FITC filter. For the evaluation, we used an excitation wavelength in the range of 450–500 nm and detection in the range of 515–565 nm. For counter staining to visualize nuclei, the specimens were mounted using UltraCruz (Santa Cruz) mounting medium with DAPI.

### 2.8. Chondrogenic Differentiation of NP-Labelled MSCs and Alcian Blue Staining

On the 13th day of culture when MSCs reached 80% confluency, CPN was added to MesenCult media in 1 : 4 ratio (25 *μ*g/mL). After MSCs were incubated with CPN for 24 h, MSCs were centrifuged for 5 min at 1500 rpm in round bottom tubes to form cell spheres. The cell spheres were induced via specific chondrogenesis induction medium composed of 10 ng/mL TGF*β* (transforming growth factor beta) 1, 100 nM dexamethasone, 50 *μ*g/mL ascorbic acid, 1 mM sodium pyruvate, 6.25 *μ*g/mL insulin, 6.25 *μ*g/mL transferrin, 6.25 *μ*g/mL selenous acid (ITS), and 1.25 mg/mL bovine serum albumin. Cell spheres were cultured for 28 days in a 5% CO_2_ incubator at 37°C. The MSCs were not further treated with CPN during differentiation. To assess the effect of CPN on chondrogenic differentiation, the cell nodules were fixed with M1 Embedding Matrix (Thermo) and Alcian Blue (Sigma) was used to detect the presence of cartilage condensations [23]. The cells were then examined under bright-field microscopy. For CPN detection, the same sections were examined under fluorescence microscopy with FITC filter. For the evaluation, we used an excitation wavelength in the range of 450–500 nm and detection in the range of 515–565 nm. For counter staining to visualize nuclei, the specimens were mounted using UltraCruz (Santa Cruz) mounting medium with DAPI.

### 2.9. *In Vivo* Tracking of NP-Labelled MSCs

Liver injury was generated by partial hepatectomy (PH) in 6-month-old Sprague Dawley rats. 70% of the liver mass was resected, and in the Sham (SH) group identical surgical procedures were performed without resection [24]. Three animals per group were used in the experiments. 10^6^ MSCs (nonlabeled) or CPN-MSCs in sterile 1x PBS were injected to the PH and SH group of animals through their tail vein. After 3 days of injection, the animals were sacrificed and their livers were removed and embedded into paraffin. 5 *μ*m thick paraffin embedded liver tissue sections were transferred to the slides. Slides were then mounted with UltraCruz mounting medium with DAPI (Santa Cruz Biotechnologies, CA, USA) for counter staining. Slides were observed with a fluorescent microscope (Leica TCS/SP5, Japan). Excitation wavelength for CPN and DAPI was at 490 nm and at 359 nm, respectively. ImageJ analysis was performed to count CPN-MSCs in the sections.

### 2.10. Statistical Analysis

All data are expressed as mean ± SD (standard deviation). Data were analyzed by performing paired *t*-test using Minitab Statistical Software (State College, PA, USA). A value of *P* < 0.05 was considered to be statistically significant.

## 3. Results

The present study was undertaken to test whether self-fluorescence nanoparticles are a valuable tool to label MSCs without affecting their marker expression, viability, and homing capacities. To accomplish this task bone marrow-derived MSCs were isolated, expanded, and labeled with fluorescent nanoparticles. The effect of labeling process on the marker expression, viability, differentiation and homing functions of MSCs were determined.

**Figure 1:**
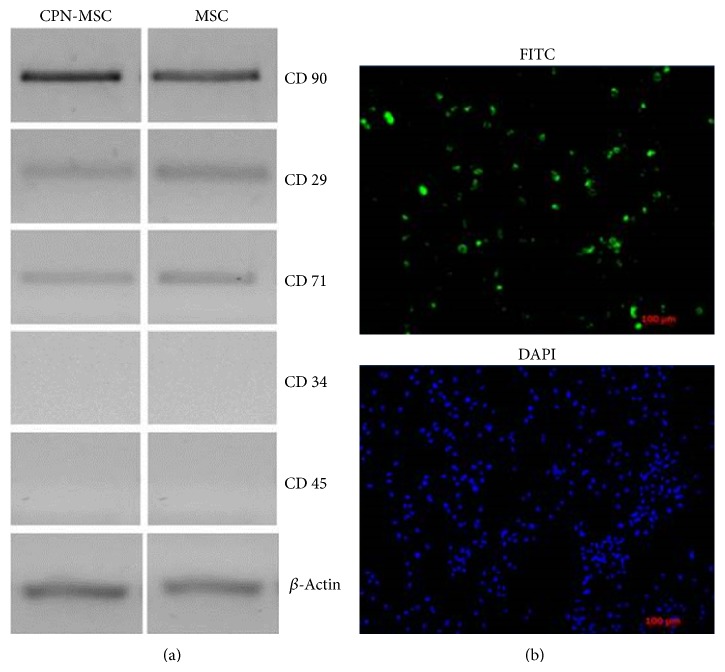
Characterization and labelling efficiency of 24 h CPN treated and nontreated MSCs. (a) After CPN-labeling MSCs (left panel) were positive for mesenchymal stem cell markers (CD90, CD29, and CD71) and negative for hematopoietic stem cell markers (CD34 and CD45) same as nontreated MSCs (right panel). *β*-Actin was used for loading control. (b) CPN-MSCs were visualized by fluorescence microscopy by using FITC filters. DAPI staining was performed to visualize cellular DNA. Magnification bar: 100 *μ*m.

In order to check whether CPN labeling has any effect on MSCs, we investigated the gene expression profiles which are characteristics of MSCs upon CPN treatment and compared to that of MSCs without CPN labelling. We investigated not only the presence of MSC markers but also the absence of hematopoietic stem cell markers. By using RT-PCR, we showed that CPN treated MSCs were positive for MSCs markers such as CD29, CD71, and CD90 and negative for hematopoietic stem cell markers such as CD34 and CD45 (Figure 1(a), left panel). The pattern of expression was the same as that of MSCs without CPN treatment (Figure 1(a), right panel). We then investigated the efficiency of CPN labeling in MSCs; our data showed that 70% of the MSCs were diffusely stained after CPN labeling measured by ImageJ analysis (Figure 1(b)).

**Figure 2:**
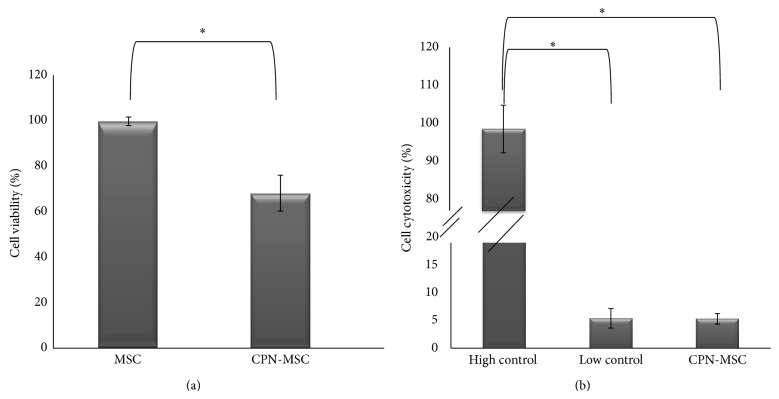
Effects of 24 h CPN labeling on MSCs' (a) cellular metabolic activity shown by MTT assay and (b) cellular toxicity shown by LDH assay (^*^ *P* < 0.005).

To assess the effect of CNPs on the viability of MSCs, we performed MTT assay. CNP-labeled MSCs retained 70% of nonlabeled control MSCs' MTT activity (Figure 2(a)). To test whether CPNs are toxic to MSCs, we performed LDH test (Figure 2(b)). It is important to note that two different controls are being used in this test. The first one is the high control where 1% Triton-X is added to the culture media to provide maximum toxicity. The second one is the low control, where no Triton-X is added, and this data provides the value for minimum toxicity. By using the formula that was given in Material and Methods section, the rate of toxicity could be calculated. Our results showed that the cytotoxicity percentage of CPN-MSC was very similar to the low control and significantly different from the high control (^*^ *P* < 0.005) suggesting that CPN to labelling is not toxic for MSCs (Figure 2(b)).

**Figure 3:**
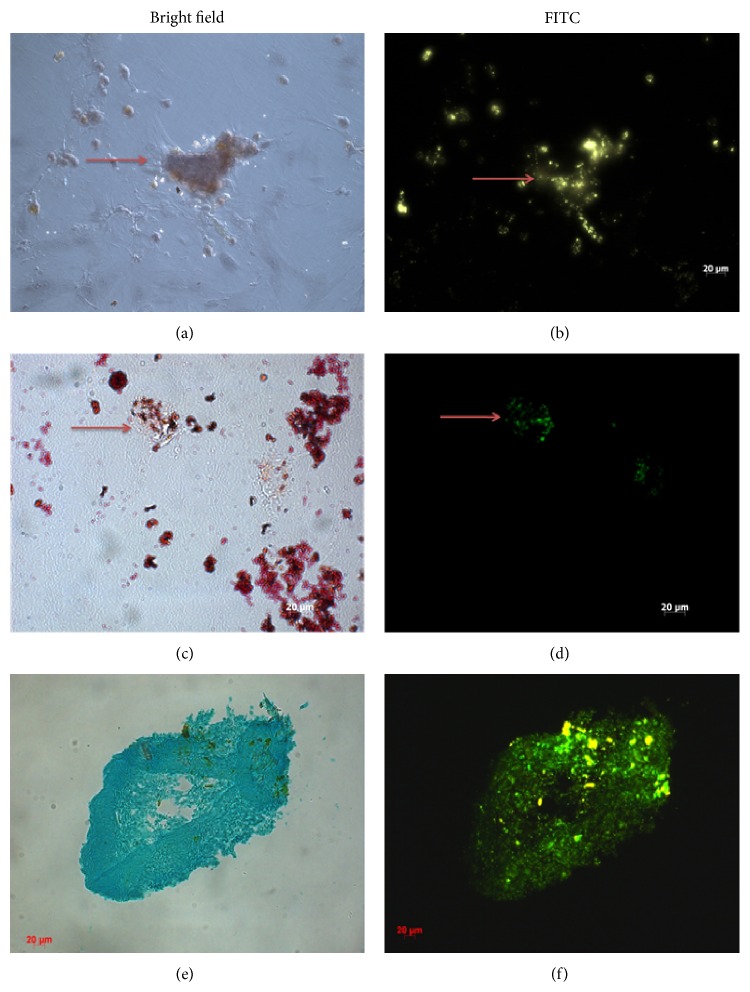
Differentiation of CPN-labeled MSCs into ((a), (b)) osteogenic lineage, ((c), (d)) adipogenic lineage, and ((e), (f)) chondrogenic lineage after culturing with induction medium. (a) Visualization of osteogenic lineage by Alizarin Red staining; (c) adipogenic lineage by Oil Red O staining; and (e) chondrogenic lineage by Alcian Blue staining under bright-field microscopy. ((b), (d), and (f)) Visualization of CPN labeling shown by using FITC filters since CPN had a similar emission and excitation wavelength to FITC. Red arrows denote some of the positive staining for the presence of CPNs after differentiation. Magnification bar: 20 *μ*m.

To evaluate whether CPN-labeled MSCs retain fluorescein signal after* in vitro* differentiation and could be used for tracking, we induced* in vitro* differentiation of MSCs into osteocytes, adipocytes, and chondrocytes after being treated with CPNs for 24 h. MSCs were cultured in osteogenic, adipogenic, and chondrogenic differentiation medium without any further CPN treatment (Figure 3). Our results showed the presence of fluorescence staining after 3 weeks for osteogenic and adipogenic differentiation (Figures 3(b) and 3(d)) and after 4 weeks for chondrogenic differentiation (Figure 3(f)). These signals were colocalized with calcium node formation shown by Alizarin Red staining (Figure 3(a)), with oil droplets shown by Oil Red O staining (Figure 3(c)), and with cartilage condensations shown by Alcian Blue staining (Figure 3(e)) under bright-field microscopy. CPN labeling intensity did not fade out after MSCs were differentiated.

**Figure 4:**
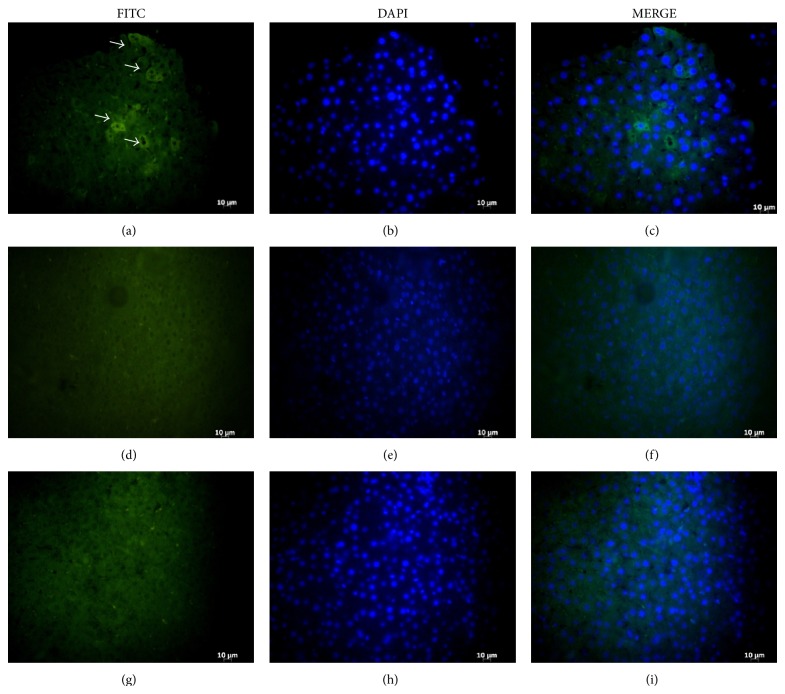
*In vivo* tracking of CPN-MSCs. Paraffin embedded liver tissues obtained from rats that were injected with either CPN-labeled ((a)–(f)) or nonlabeled MSCs ((g)–(i)) from tail vein followed by PH ((a)–(c) and (g)–(i)) and SH ((d)–(f)). White arrows denote injected CPN-labelled MSCs under FITC filter. Magnification bar: 10 *μ*m.

To validate the use of CPN-MSCs, we used a widely accepted model of liver injury model. Rats underwent either partial hepatectomy (PH) operation to induce injury (Figures 4(a)–4(c) and 4(g)–4(i)) or Sham (SH) operation for control (Figures 4(d)–4(f)). Then these animals were injected either with CNP-MSCs (Figures 4(a)–4(f)) or nonlabeled MSCs (Figures 4(g)–4(i)) from tail vein. Three days after the injection, the rats were sacrificed and their livers were sectioned and analyzed with fluorescence microscopy. Our results showed that several CPN-MSCs were present when these cells were injected after PH (Figures 4(a)–4(c)). ImageJ analysis revealed that 8% of the cells in the section were CPN labelled. No staining was observed in the liver sections after CPN-labeled MSCs were injected to SH group (Figures 4(d)–4(f)) or when nonlabeled MSCs were injected to PH group (Figures 4(g)–4(i)) as expected.

## 4. Discussion

MSCs have a high potential for regenerative medicine and tissue engineering not only due to their intrinsic self-renewal capacity and ability to differentiate functional cell types in specific tissues but also due to their homing capacity and nonimmunogenic features [1, 2, 7]. Since MSCs are important tools for cell-based therapies [25–27] and nanoparticles are promising labeling tools in noninvasive cell tracking [28], complete assessment of CPNs on stem cells should be performed.

First of all, the labeling material should be safe and not toxic as well as easy to use. It was reported that use of nanoparticles may cause cytotoxic effect on the cell [10] and this has to be evaluated and this evaluation may not be very straightforward [29]. This assessment should be performed individually for each nanoparticle and cell type. We used MTT and LDH assay as reliable quantitative methods to assess the effect of CPNs on rat bone marrow-derived MSCs, since these assays have been demonstrated as sensitive, precise, convenient, rapid, and economical test method by many studies for the measurement of* in vitro* cytotoxicity, cell adhesion, cell proliferation, and cell number [30, 31]. It has been shown that at the MTT results if the cell viability is less than 40%, the tested material would be toxic and should be further investigated with other methods [32]. When we performed cytotoxicity testing 24 hours after CPN labelling, the MTT survival rate was 70% for the CPN-labeled cells and therefore should not be considered as toxic. In addition, LDH assay revealed that CPN treated MSCs behaved very similar to that of MSCs alone (low control), further suggesting the safety of CPN labeling. In addition, CPN's absorbance and emission characteristics were similar to FITC which could easily be monitored.

Other than cytotoxicity, labeling agent's effect on the markers of MSCs is also an important point. Therefore, the expression profiles of CD90, CD71, CD29, CD34, and CD45 were tested to identify whether CPN treatment affected the characteristics of MSCs. As shown previously by us and others, these markers are widely used to characterize MSCs [4, 8, 9]. CPN-labeled MSCs expressed CD90, CD71, and CD29 but not CD34 or CD45, suggesting that CPN labeling did not affect these cells' characteristics. In addition incubation of MSCs with CPN for 24 h resulted in stably labeled MSCs with diffuse cytoplasmic staining.

The retaining of the label during the stem cell differentiation is also a critical issue. This is especially important in MSC biology because current* in vitro* regimen for differentiation takes three weeks for osteogenic and adipogenic differentiation and four weeks for chondrogenic differentiation. When CPN-MSCs were cultured in osteogenic, adipogenic, and chondrogenic differentiation medium for three to four weeks without any further CPN addition, they differentiated with their fluorescent labels intact. There was no effect of nanoparticles on the osteogenic, adipogenic, and chondrogenic differentiation ability of mesenchymal stem cells since CPN-MSCs showed the characteristic stainings of osteogenic, adipogenic, and chondrogenic lineages by Alizarin Red, Oil Red O, and Alcian Blue, respectively. These stainings have been used to assess the differentiation for non-labelled, control MSCs by us and others [9, 23].

To be able to track cells* in vivo* is critical to better understand the biology during their migration and function. A great deal of attention has been given to understand what regulates MSCs' migration to an injury site. However, tracking cells within animal's body is a compulsive process. Many attempts to track and monitor stem cells and cancer cells* in vivo* (such as by quantum dot labeling, SPOI labeling, and MRI detection) are challenged by cellular toxicity caused by these materials, inadequate duration of labeling, and alteration of gene expression or cellular functions [33–35]. Promisingly, our results showed that CPN labeling provides an opportunity to track MSCs* in vivo* safely, since labeled MSCs migrated to injured liver and resided there without losing their labels. However, whether these CPN-MSCs differentiate in the injured liver is a valid question and further experiments to test this are being planned. It is also important to note the possibility of label dilution following MSC proliferation longer-term* in vitro* and potentially* in vivo*, as it could contribute to the rate of detection of fluorescent cells on liver sections.

## 5. Conclusion

We report a new and safe way to track stem cells* in vitro* and* in vivo*. CPN labeling might be a potential tool for the tracking of MSCs and therefore can be used to better understand MSCs' differentiation and homing mechanisms without any manipulations performed at the DNA level such as GFP or luciferase labeling.

## Figures and Tables

**Table 1:** RT-PCR conditions for each primer.

Beta-actin	Forward Reverse	5′-CTGGCCTCACTGTCCACCTT-3′ 5′-GGGCCGGACTCATCGTACT-3′	65 bp

CD90	Forward Reverse	5′-CCAGTCATCAGCATCACTCT-3′ 5′-AGCTTGTCTCTGATCACATT-3′	374 bp

CD34	Forward Reverse	5′-TGTCTGCTCCTTGAATCT-3′ 5′-CCTGTGGGACTCCAACT-3′	281 bp

CD71	Forward Reverse	5′-ATGGTTCGTACAGCAGCAGA-3′ 5′-CGAGCAGAATACAGCCATTG-3′	182 bp

CD29	Forward Reverse	5′-ACTTCAGACTTCCGCATTGG-3′ 5′-GCTGCTGACCAACAAGTTCA-3′	190 bp

CD45	Forward Reverse	5′-ATGTTATTGGGAGGGTGCAA-3′ 5′-AAAATGTAACGCGCTTCAGG-3′	175 bp

**Table 2:** RT-PCR conditions for each primer.

Genes	Initial denaturation	Denaturation	Annealing	Extension	Cycle	Final extension
Beta-actin	95°C, 5 min	94°C 40 sec	60°C 35 sec	72°C 40 sec	25	72°C, 5 min
CD90	95°C, 5 min	94°C 30 sec	55°C 30 sec	72°C 30 sec	30	72°C, 5 min
CD34	95°C, 5 min	94°C 30 sec	55°C 30 sec	72°C 30 sec	30	72°C, 5 min
CD71	95°C, 5 min	94°C 40 sec	66°C 60 sec	72°C 40 sec	35	72°C, 5 min
CD29	95°C, 5 min	94°C 30 sec	60°C 30 sec	72°C 30 sec	29	72°C, 5 min
CD45	95°C, 5 min	94°C 30 sec	60°C 30 sec	72°C 30 sec	23	72°C, 5 min

**Table 3:** The raw data for MTT assay.

	MSC %	CPN-MSC %
Sample 1	97.95	71.46
Sample 2	101.03	59.14
Sample 3	101.03	73.92
Standard deviation (SD)	1.78	7.92

**Table 4:** The raw data for LDH assay.

	High control %	Low control (MSC) %	MSC-CPN %
Sample 1	94.08	10.79	4.45
Sample 2	100.22	5.90	6.29
Sample 3	105.71	8.18	5.98
Standard deviation (SD)	5.82	2.45	0.99
